# The collaboration and reporting quality of social welfare systematic reviews in the Campbell Collaboration online library

**DOI:** 10.1186/s12955-019-1241-7

**Published:** 2019-11-07

**Authors:** Li-li Wei, Jing Zhang, Ying Yang, Hao-Yu Cao, Ke-hu Yang, Li-Juan Si, Jin-Hui Tian

**Affiliations:** 1 School of Economics, Lanzhou University, 222 Tianshui South Ave., 730000 Lanzhou, Gansu People’s Republic of China; 2 Evidence-based Social Science Research Center, Lanzhou University, 222 Tianshui South Ave., Lanzhou, Gansu 730000 People’s Republic of China; 3 Evidence-based Medicine Center, Lanzhou University, 222 Tianshui South Ave., 730000 Lanzhou, Gansu People’s Republic of China

**Keywords:** Reporting quality, Social welfare, Campbell collaboration

## Abstract

**Background:**

To analyze the collaboration and reporting quality of the systematic reviews of social welfare in the Campbell collaboration online library.

**Methods:**

The Campbell collaboration online library was searched for systematic reviews of social welfare and the basic information extracted in order to assess the reporting quality of systematic reviews using a MOOSE checklist. BICOMS-2 and UCINET software were used to produce the social network, and Comprehensive Meta Analysis (Version 2) and STATA 13.0 were used to analyze the related data.

**Results:**

Fifty-seven systematic reviews of social welfare were included. Twenty-eight items of the included social welfare systematic reviews were rated as complete (≥70%). There were significant differences between ≤2013 and ≥ 2014 in five items. These differences were as follows: research published by one organization or more than one organization in one item, more than three authors or less than four authors in two items, and one country or more than one country in six items. It’s completed about researches with more than one organization, three authors or more than one country. Some items were found to have a low reporting rate of studies published before 2014, by one organization, with less than four authors or one country, respectively. The social network of authors and organizations showed good collaboration.

**Conclusions:**

Some items could be further improved with regard to the rate of reporting systematic reviews of social welfare in the Campbell collaboration online library. This could improve the overall quality of social welfare systematic reviews.

## Background

In recent years, the rigorous concepts and methods of evidence-based medical science have rapidly begun to penetrate and be applied to other disciplines. Compared with the “scientific” process of the natural sciences, the “scientific” process in the social sciences is relatively lagging behind. The systematic reviews evidence database established by the Campbell Collaboration Network in 2000 aims to promote evidence-based concepts and methods in the fields of education, justice, welfare, and international development [1, 2]. It also promotes the application and development of evidence-based practice in the fields of society, education, psychology, law, economics and management [3–5].

Social welfare refers to the funds and social security system provided by a country that guarantee a certain standard of living and aim to maximize the quality of life. Social welfare is also a regulator of social contradictions. Social welfare services generally include the following: medical and health care services, cultural and educational services, labor and employment services, residential services, isolated and disabled services, disability rehabilitation services, crime correction and probation services, mental health services, and public welfare services [6–9]. The application of Campbell’s systematic reviews in the field of social welfare has focused on social interventions, and social welfare public policies. Social welfare systematic reviews provide a comprehensive assessment of the available evidence, and high-quality research evidence for decision-making. These systematic reviews also focus on the effectiveness of macro policy interventions and analyze factors that may affect welfare outcomes.

The Campbell Library is the main product of the Campbell Collaboration, and is internationally recognized for its comprehensiveness and for maintaining the highest standards in evidence-based social science. So far, 158 Campbell reviews and 223 protocols have been published online in the Campbell Library, alongside many social welfare systematic reviews (SWSRs). SWSRs could help policy makers to improve the quality of social welfare.

In order to select high-quality research, the current study applies a checklist [10–14] from the medical observational research field when considering the reporting quality of SWSRs. In light of this, the aims of the current study were to assess the reporting quality of SWSRs and perform a subgroup analysis of the factors affecting this quality. The collaboration of authors and organizations is also analyzed.

## Methods

### Search strategy, inclusion and exclusion criteria

We have browsed the website of the Campbell collaboration online library (https://www.campbellcollaboration.org/library.html). SWSRs were included that met the following criteria: First, those that synthesized evidence in the classification of social welfare, including the formulation, implementation and evaluation of social welfare and research methods; Second, those containing complete information, including author related information, funding, and time of publication; Third, those that were available in the library on 29 March 2018, when all of the relevant studies were downloaded. It should be noted that only systematic reviews with complete information were included. The protocol and title files for the Campbell Library were excluded.

### Study selection process and data extraction

Firstly, two investigators independently screened the acquired the SWSRs. Secondly, one investigator (Ying Yang) downloaded the original full text SWSRs. Thirdly, using a data extraction sheet, two reviewers (Jin-Hui Tian and Jing Zhang) independently extracted the SWSRs that met the stipulated characteristics. Following this, a standard form table was constructed in order to extract the guideline data, including the time of publication, authors’ countries and organizations, funding, and key study information such as content, methods and results. Two reviewers (Jin-Hui Tian and Jing Zhang) extracted the data separately, with any disagreements discussed or with a third reviewer (Li-Juan Si) if no consensus had been reached.

### Quality assessment

The reporting quality of systematic reviews reflects the standard and risk of bias or validity in its processes and results [15]. The reporting quality of the SWSRs was evaluated using the MOOSE checklist, which includes the following six quality-related sections: background, search strategy, methods, results, discussion and conclusion. There are 35 question items in six sections. In order to assess the quality of the reviews, the assessor needs to respond to the 35 questions for each SWSR, with a “yes”, “partial”, or “no”. The total reporting quality score can then be obtained by summing 1 point for each “yes”, 0.5 for each “partial”, and 0 points for any other responses (“no” or “cannot answer”), with a maximum total score of 35. The scores were ranked into three groups: low quality (≤20 points), medium quality (21–27 points), and high quality (above 27 points).

### Statistical analysis

Clearly showing the overall characteristics of SWSRs can provide direction for researchers and policymakers. The authors’ and organizations’ social networks were produced using BICOMS-2 (Bibliographic Item Co-Occurrence Mining System) and Netdraw in UCINET. The reporting rate and 95% confidence interval of each item were extracted using Comprehensive Meta Analysis (Version 2). To begin with, the relevant information was extracted, including the number of authors, organizations and countries, and the text file was constructed following the BICOMS-2 format. The file was then imported into the software and the collinear matrix produced. Next, the descriptive tables and figures were produced following the quantity and frequency calculation. The subgroup analysis was undertaken alongside this, which included the year of publication, the number of organizations, the number of authors and the number of countries involved in each study. STATA13.0 was used to analyze the relevant data and to produce the odds ratio and 95% confidence interval of each subgroup. Statistical significance was defined as two-sided *P* < 0.05.

## Results

The website of the Campbell Library was screened for related researches. The final sample included 57 SWSRs.

### Distribution of time of publication

**Fig. 1:**
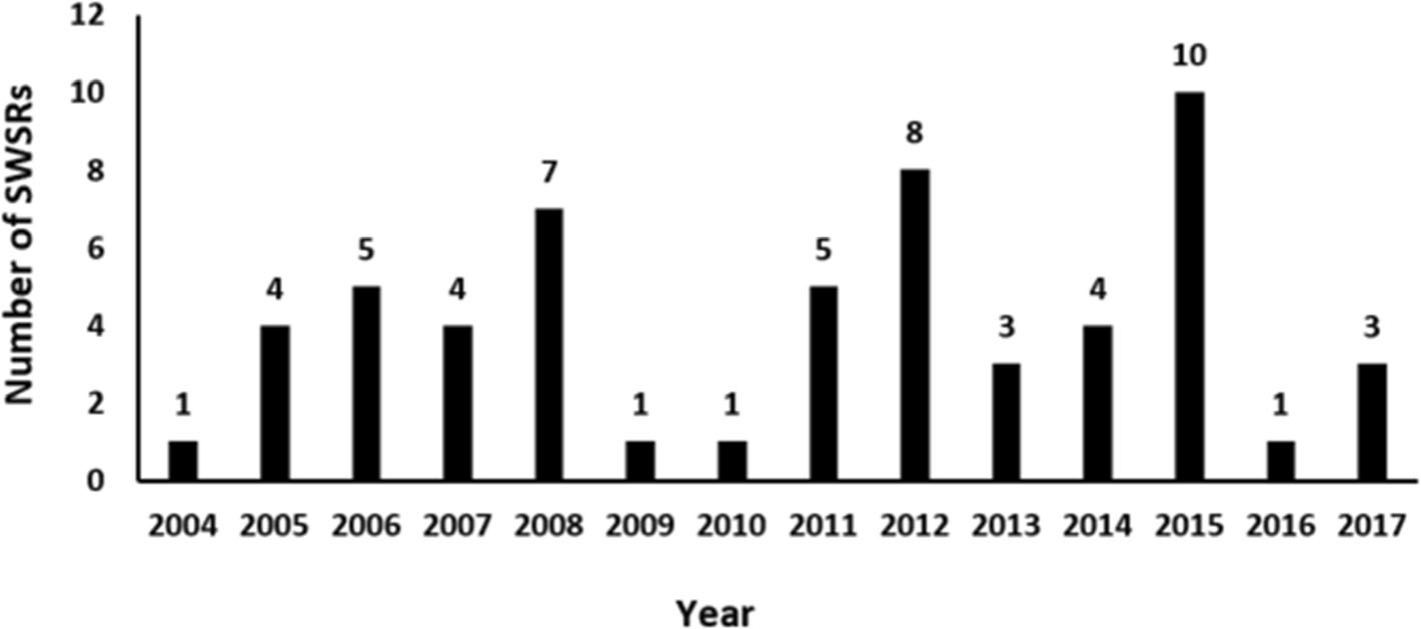
Distribution of time of publication

The first SWSR was published by the Social Welfare group on the Campbell Library in 2004. From 2004 to 2017, the publishing trend was not stable. In particular, 2015 saw a peak of publications, with 10 SWSRs being published that year. However, the overall number of published SWSRs was low, under 15 per year. These results are shown in Fig. 1.

### Country distribution

**Fig. 2:**
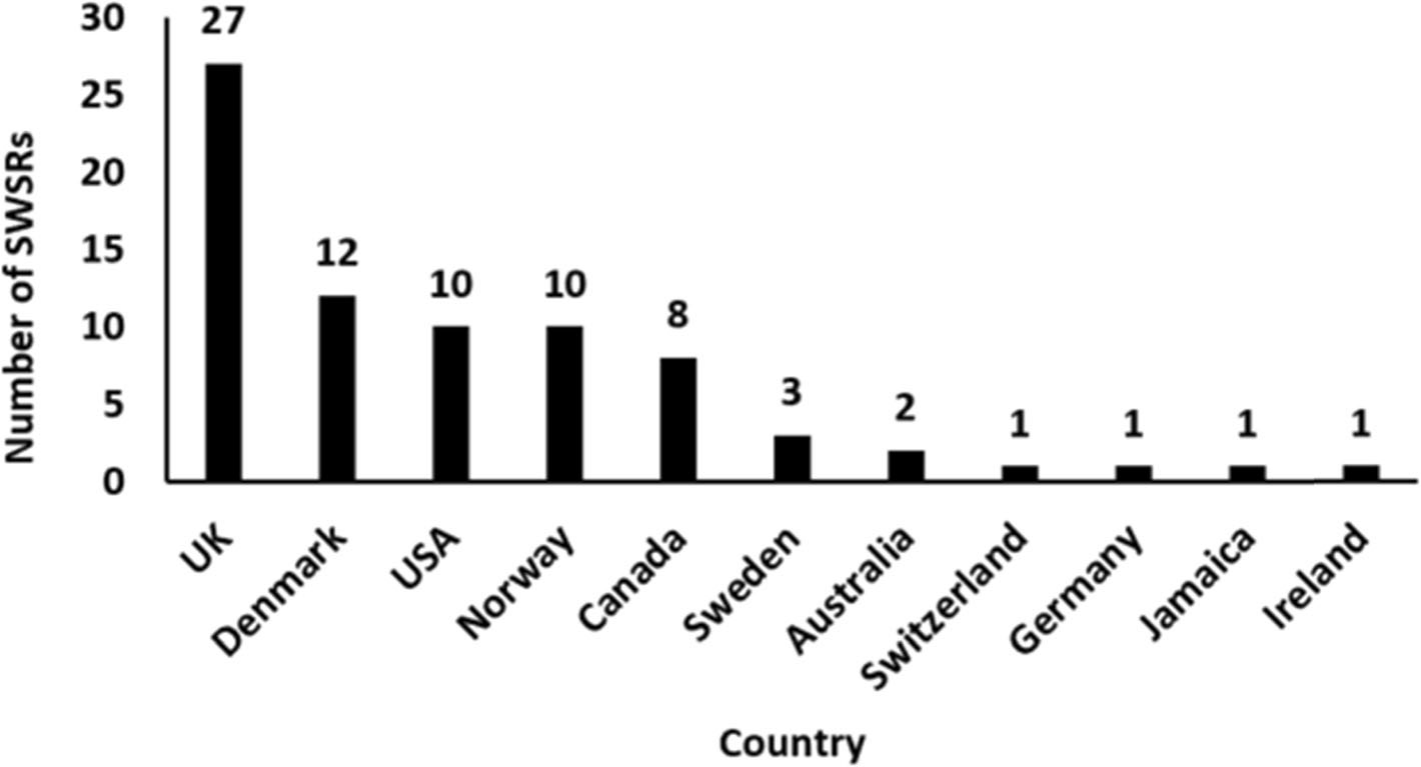
Country distribution

Authors from 11 countries published SWSRs under the Social Welfare group on the Campbell Library. Out of the 57 social welfare systematic reviews studied, 27 authors from the UK accounted for 35.5% of all authors. Twelve authors from Denmark accounted for 15.8%, 10 from the USA accounted for13.2%, and 10 from Norway also accounted for 13.2%. The highest number of SWSRs was found to have been published by authors from the UK, far more than those from other countries. Moreover, SWSRs were almost all published by authors from developed countries, with the exception of Jamaica (Fig. 2). The former has made great contributions to the research in this field, while research in underdeveloped countries needs to be strengthened. Most of the SWSRs authors belonged to the SFI Campbell organization, meaning that there were more authors from Denmark.

### Distribution and collaboration of the authors

In total, there were found to be 129 authors involved in the included SWSRs. Table 1 shows the 21 authors who were involved with more than one SWSR. Among the 129 authors, 108 (83.7%) were involved with one SWSR, 12 (9.3%) with 2–3 SWSRs, five (3.9%) with 4–5 SWSRs, and three (2.3%) with 6–9 SWSRs. Only Trine Filges had participated in 10 SWSRs. These findings reveal that while these authors have long been engaged in social welfare-related research, there were not many high-yielding authors. Many authors (83.7%) had only published one SWSR. Authors from SFI Campbell tended to have published more SWSRs.

**Table 1:** Authors who had published more than one SWSR

Author (Organization)	N (%)
Trine Filges (SFI Campbell)	10 (17.5)
Paul Montgomery (University of Oxford)	9 (15.8)
Anne Marie Klint Jorgensen (SFI Campbell)	8 (14.0)
Jane A Dennis (University of Bristol)	6 (10.5)
Krystyna Kowalski (SFI Campbell)	5 (8.8)
Evan Mayo-Wilson (University of Oxford)	4 (7.0)
Jane Barlow (University of Warwick)	4 (7.0)
Geir Smedslund (Norwegian Knowledge Centre for the Health Services)	4 (7.0)
Maia Lindstrom (SFI Campbell)	4 (7.0)
Geraldine Macdonald (Queen’s University Belfast)	3 (5.3)
Pernille Skovbo Rasmussen (SFI Campbell)	3 (5.3)
Asbjorn Steiro (Norwegian Knowledge Centre for the Health Services)	2 (3.5)
Frances Gardner (University of Oxford)	2 (3.5)
Marc Winokur (Colorado State University)	2 (3.5)
Lars Pico Geerdsen (SFI Campbell)	2 (3.5)
William Turner (University of Bristol)	2 (3.5)
Sabine Wollscheid (Norwegian Knowledge Centre for the Health Services)	2 (3.5)
Herrick Fisher (University of Oxford)	2 (3.5)
Mark Petticrew (The London School of Hygiene & Tropical Medicine)	2 (3.5)
Hannah Jones (University of Bristol)	2 (3.5)
Ditte Andersen (SFI Campbell)	2 (3.5)

**Fig. 3:**
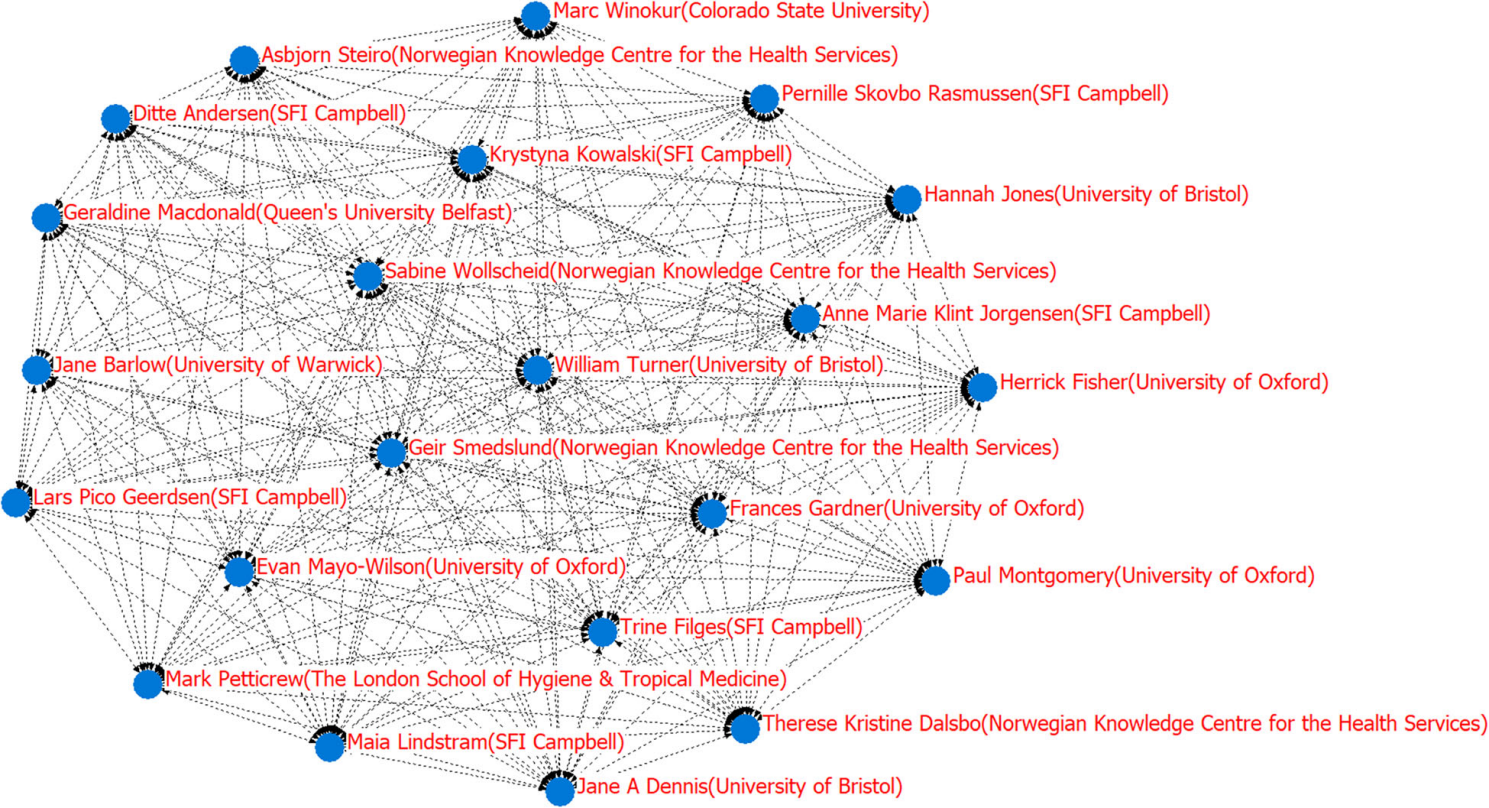
Authors’ social network

Figure 3 shows the social network of the authors of the examined SWSRs. Here, and with reference to the 21 authors shown in Table 1, a 21*21 co-occurrence matrix produced by Netdraw software was used to show their social network relationships. The high-yielding authors at the forefront (Table 1) can be found at the edge of the network, including Trine Filges (SFI Campbell), Paul Montgomery (University of Oxford), Anne Marie Klint Jorgensen (SFI Campbell), and Jane A Dennis (University of Bristol),and have a low level of links with other authors who have not formed clear-cut research groups and networks. To a certain extent, this inconsistency will arguably slow down the research progress of evidence-based social welfare. In future research, researchers should strengthen cooperation in order to promote the development of evidence-based social welfare research. William Turner (University of Bristol), Herrick Fisher (University of Oxford), Krystyna Kowalski (SFI Campbell), and Sabine Wollscheid (Norwegian Knowledge Centre for the Health Services) are located in the center of the social network and appeared most often in the same SWSRs with other authors; to an extent, this indicates that their studies may reflect topical research in the SWSR field.

### Organization distribution and collaboration

Sixty-five organizations were found to be involved in the included SWSRs. Table 2 shows the 15 organizations that were involved with more than one SWSR, highlighting that SFI Campbell and the University of Oxford published over 10 SWSRs. Overall, almost all of the authors were from universities and international welfare and health organizations. As the main research institutions, universities were found to be the site where most of the researchers were concentrated, and the connections between the researchers emerged as strong. Many original types of research have laid a good foundation for SWSRs. International welfare and health organizations are more inclined to be engaged in practice and have a very good understanding of the implementation of social welfare, which provides a good practical basis for SWSRs.

**Table 2:** Organizations in which more than one SWSR were published

Organization	N (%)
SFI Campbell	11 (19.3)
University of Oxford	11 (19.3)
University of Bristol	9 (15.8)
Norwegian Knowledge Centre for the Health Services	8 (14.0)
Queen’s University Belfast	5 (8.8)
University of Warwick	4 (7.0)
University of Ottawa	4 (7.0)
Colorado State University	3 (5.3)
The Centre for Child and Adolescent Mental Health	2 (3.5)
University of London	2 (3.5)
The London School of Hygiene & Tropical Medicine	2 (3.5)
International Labour Organization	2 (3.5)
University of Toronto	2 (3.5)
City University	2 (3.5)
Vanderbilt University	2 (3.5)

**Fig. 4:**
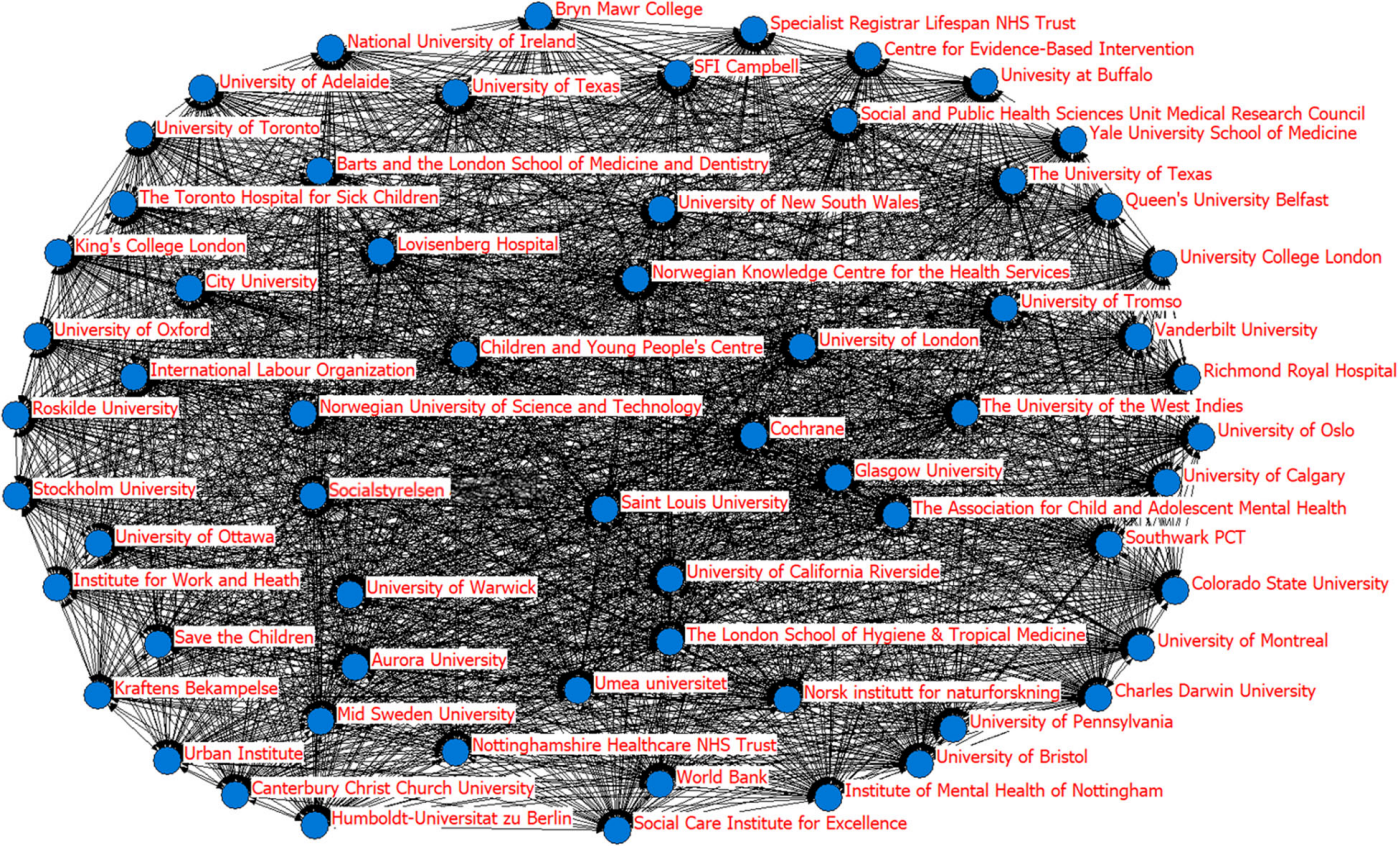
Organizations’s social network

Figure 4 shows the social network of the organizations that had published the SWSRs, with all of the organizations included. A 65*65 co-occurrence matrix constructed using Netdraw software was used to produce social network relationships. Some higher-frequency organizations were found to have less cooperation with other agencies, such as SFI Campbell, the University of Oxford, the University of Bristol, and the Norwegian Knowledge Centre for the Health Services. The University of Warwick, the Urban Institute, Barts and the London School of Medicine and Dentistry, Colorado State University, Socialstyrelsen, the University of London and Oslo University College were located in the center of the social network and appeared most often in the same SWSRs with other organizations, indicating that their research may, to a certain extent, reflect key topical study areas in the field of SWSRs.

### Reporting quality of SWSRs

Table 3 shows the results pertaining to reporting quality as yielded by the MOOSE checklist. Twenty-eight items were rated as complete (≥70%) and six items in the background as relatively complete. All of the SWSRs’ abstracts were found to be structured, adequately stating the study background, methods, reported data sources and selection criteria, and ending with a conclusion that summarized the review’s main findings. As part of the Campbell Library’s reporting standard, all systemic reviews need to report their search strategy, and include their exclusion criteria and database selection, in order to ensure their quality. However, the current study’s search strategy reveals that most of the SWSRs shown in Table 3 did not completely report qualification (66.7%), research information (59.7%) and language (66.7%), and that retrieval software (0) yielded almost no reports.

**Table 3:** Results of reporting quality according to MOOSE checklist [%(95%CI)]

Item		Yes	Partial	No
Background	Research questions	[100 (93.70, 100.00)]	[0 (0.00, 6.31)]	[0 (0.00, 6.31)]
	Research hypothesis	[100 (93.70, 100.00)]	[0 (0.00, 6.31)]	[0 (0.00, 6.31)]
	Outcomes	[100 (93.70, 100.00)]	[0 (0.00, 6.31)]	[0 (0.00, 6.31)]
	Types of interventions	[100 (93.70, 100.00)]	[0 (0.00, 6.31)]	[0 (0.00, 6.31)]
	Types of study	[100 (93.70, 100.00)]	[0 (0.00, 6.31)]	[0 (0.00, 6.31)]
	Population	[98.3 (90.63, 99.99)]	[0 (0.00, 6.31)]	[1.8 (0.00, 9.42)]
Search strategy	Qualification	[29.8 (18.40, 43.43)]	[3.5 (0.41, 12.13)]	[66.7 (52.90, 78.61)]
	Strategy	[94.7 (85.42, 98.90)]	[1.8 (0.00, 9.42)]	[3.5 (0.41, 12.13)]
	Research information	[40.4 (27.38, 54.23)]	[59.7 (45.80, 72.41)]	[0 (0.00, 6.31)]
	Electronic searches	[96.5 (87.91, 99.57)]	[0 (0.00, 6.31)]	[3.5 (0.41, 12.13)]
	Retrieval software	[0.0 (0.00, 6.31)]	[0 (0.00, 6.31)]	[100.0 (93.70, 100.00)]
	Inclusion and exclusion criteria	[45.6 (32.43, 59.31)]	[00 (0.00, 6.31)]	[54.4 (40.71, 67.60)]
	Hand searching	[89.5 (78.53, 96.01)]	[8.8 (2.90, 19.31)]	[1.8 (0.00, 9.42)]
	Language	[24.6 (14.1, 37.80)]	[66.7 (52.90, 78.61)]	[8.8 (2.90, 19.31)]
	Content incompleteness	[57.9 (44.10, 70.91)]	[40.4 (27.61, 54.20)]	[1.8 (0.00, 9.42)]
	Personal contacts	[77.1 (64.21, 87.30)]	[22.8 (12.68, 35.79)]	[0 (0.00, 6.31)]
Methods	Literature correlation	[98.3 (90.61, 99.99)]	[1.8 (0.00, 9.42)]	[0 (0.00, 6.31)]
	Quantitative Data Synthesis	[100.0 (93.70, 100.00)]	[0.0 (0.00, 6.31)]	[0 (0.00, 6.31)]
	Blinding	[98.3 (90.63, 99.99)]	[0.0 (0.00, 6.31)]	[1.8 (0.00, 9.42)]
	Confounding	[52.6 (39.01, 66.12)]	[0.0 (0.00, 6.31)]	[47.3 (34.00, 61.03)]
	Regression analysis	[98.3 (90.61, 99.99)]	[1.8 (0.00, 9.42)]	[0 (0.00, 6.31)]
	Heterogeneity	[98.3 (90.61, 99.99)]	[0 (0.00, 6.31)]	[1.8 (0.00, 9.42)]
	Model description	[98.3 (90.61, 99.99)]	[0 (0.00, 6.31)]	[1.8 (0.00, 9.42)]
	Appropriate charts	[84.2 (72.10, 92.53)]	[0 (0.00, 6.31)]	[15.8 (7.46, 27.89)]
Results	Table display	[94.7 (85.41, 98.90)]	[0 (0.00, 6.31)]	[5.3 (1.13, 14.61)]
	Chart display	[100 (93.70, 100.00)]	[0 (0.00, 6.31)]	[0 (0.00, 6.31)]
	Sensitivity analysis	[96.5 (87.89, 99.59)]	[0 (0.00, 6.31)]	[3.5 (0.41, 12.13)]
	Uncertainty of results	[100 (93.70, 100.00)]	[0 (0.00, 6.31)]	[0 (0.00, 6.31)]
Discussion	Potential biases	[100 (93.70, 100.00)]	[0 (0.00, 6.31)]	[0 (0.00, 6.31)]
	Rationality of exclusion criteria	[84.2 (72.10, 92.53)]	[15.8 (7.46, 27.89)]	[0 (0.00, 6.31)]
	Quality of included studies	[100.0 (93.70, 100.00)]	[0 (0.00, 6.31)]	[0 (0.00, 6.31)]
Conclusion	Other reasons for the result	[96.5 (87.89, 99.59)]	[0 (0.00, 6.31)]	[3.5 (0.41, 12.13)]
	Extension of results	[98.3 (90.61, 99.99)]	[0 (0.00, 6.31)]	[1.8 (0.00, 9.42)]
	Implications	[98.3 (90.61, 99.99)]	[0 (0.00, 6.31)]	[1.8 (0.00, 9.42)]
	Funding	[96.5 (87.89, 99.59)]	[0 (0.00, 6.31)]	[3.5 (0.41, 12.13)]

The methodological aspect of these SWSRs were found to be relatively complete, as most authors included a detailed description of quantitative data synthesis, blinding, regression analysis, heterogeneity, and the models they developed. However, confounding (52.6%) was inadequately reported. In addition, while the results of the item regarding whether appropriate charts were used to illustrate the content of the paper (84.2%) were relatively complete, the report was less adequate than that pertaining to other items. A summary of the review’s key findings and suggestions for future research were found to be adequately reported in the results and discussion section. However, discussions of the rationale behind exclusion criteria (84.2%), other reasons for the results (96.49%), sensitivity analyses (96.5%) and funding (96.5%) were found to require further improvement.

### Subgroup analysis of the reporting quality of SWSRs

#### Year of publication

Table 4 shows the emergence of significant differences between ≤2013 and ≥ 2014 in terms of five items, which included research information [OR = 6.00, 95%CI (1.84, 19.53)], inclusion and exclusion criteria [OR = 4.00, 95%CI (1.28, 12.53)], language [OR = 0.25, 95%CI (0.08, 0.83)], blinding [OR = 47.91, 95%CI (2.70, 851.15)] and confounding [OR = 0.05, 95%CI (0.01, 0.41)]. At the same time, compared with the SWSRs published before 2014, 11 items (population, strategy, electronic searches, hand searches, language, personal contacts, literature correlation, confounding, rationality of exclusion criteria, other reasons for the results and funding), were found to have a high rate of reporting after 2013.

**Table 4:** Subgroup analysis of reporting quality by MOOSE checklist [OR (95%CI)]

Item		Published year	Number of organizations	Number of authors	Number of countries
		≤2013 (*n* = 36) vs ≥2014 (*n* = 21)	one (*n* = 29) vs ≥2 (*n* = 28)	≤3 (*n* = 37) vs ≥4 (*n* = 20)	one (*n* = 45) vs ≥2 (*n* = 12)
Background	Research questions	Not estimated	Not estimated	Not estimated	Not estimated
	Research hypothesis	Not estimated	Not estimated	Not estimated	Not estimated
	Outcomes	Not estimated	Not estimated	Not estimated	Not estimated
	Types of interventions	Not estimated	Not estimated	Not estimated	Not estimated
	Types of study	Not estimated	Not estimated	Not estimated	Not estimated
	Population	0.19 (0.01, 4.81)	3.22 (0.13, 82.38)	0.59 (0.02, 15.25)	11.87 (0.45, 310.85)
Search strategy	Qualification	1.85 (0.58, 5.89)	0.81 (0.27, 2.44)	0.48 (0.15, 1.55)	0.51 (0.14, 1.91)
	Strategy	0.27 (0.02, 3.19)	8.10 (0.40, 164.32)	4 (0.34, 47.11)	**33.53 (1.60, 704.07)**
	Research information	**6 (1.84, 19.53)**	1.46 (0.50, 4.24)	**0.20 (0.06, 0.64)**	0.61 (0.17, 2.19)
	Electronic searches	0.57 (0.03, 9.64)	1.04 (0.06, 17.43)	0.35 (0.02, 7.57)	**0 (0.00, 0.04)**
	Retrieval software	1.75 (0.10, 29.53)	0.18 (0.01, 3.91)	**684 (40.49, 12000)**	4 (0.79, 20.38)
	Inclusion and exclusion criteria	**4 (1.28, 12.53)**	1.66 (0.58, 4.74)	0.41 (0.13, 1.24)	**14.35 (3.08, 66.93)**
	Hand searching	0.55 (0.10, 2.99)	1.26 (0.21, 7.64)	0.92 (0.15, 5.50)	**0.06 (0.01, 0.30)**
	Language	**0.25 (0.08, 0.83)**	140 (0.41, 4.71)	0.29 (0.08, 1.01)	2.74 (0.72, 10.43)
	Content incompleteness	1.42 (0.38, 5.33)	1.90 (0.66, 5.51)	0.68 (0.18, 2.51)	1.76 (0.47, 6.56)
	Personal contacts	0.05 (0.00, 1.05)	**0.05 (0.01, 0.44)**	0.18 (0.01, 3.55)	Not estimated
Methods	Literature correlation	0.19 (0.01, 4.81)	3.22 (0.13, 82.38)	5.77 (0.22, 148.35)	11.87 (0.45, 310.85)
	Quantitative Data Synthesis	1.82 (0.07, 46.63)	Not estimated	Not estimated	1.19 (0.05, 30.96)
	Blinding	**47.91 (2.70, 851.15)**	0.33 (0.01, 8.53)	0.6 (0.02, 15.25)	**0.04 (0.00, 0.69)**
	Confounding	**0.05 (0.01, 0.41)**	0.61 (0.21, 1.74)	1.45 (0.45, 4.69)	**22 (2.17, 223.23)**
	Regression analysis	1.82 (0.07, 46.63)	0.33 (0.01, 8.53)	0.6 (0.02, 15.25)	1.19 (0.05, 30.96)
	Heterogeneity	1.82 (0.07, 46.63)	3.22 (0.13, 82.38)	0.6 (0.02, 15.25)	1.19 (0.05, 30.96)
	Model description	14.86 (0.82, 269.77)	3.22 (0.13, 82.38)	0.6 (0.02, 15.25)	0.15 (0.01, 2.84)
	Appropriate charts	4.49 (0.22, 91.35)	1.36 (0.33, 5.69)	0.48 (0.09, 2.55)	0.49 (0.02, 10.05)
Results	Table display	Not estimated	8.1 (0.40, 164.32)	0.24 (0.01, 4.89)	Not estimated
	Chart display	3.12 (0.14, 68.05)	Not estimated	Not estimated	0.7 (0.03, 15.46)
	Sensitivity analysis	Not estimated	1.04 (0.06, 17.43)	1.9 (0.11, 32.01)	Not estimated
	Uncertainty of results	Not estimated	Not estimated	Not estimated	Not estimated
Discussion	Potential biases	10.93 (0.59, 201.92)	Not estimated	Not estimated	0.21 (0.01, 3.86)
	Rationality of exclusion criteria	0.11 (0.01, 2.34)	0.46 (0.10, 2.06)	0.91 (0.20, 4.11)	21.67 (0.97, 485.61)
	Quality of included studies	Not estimated	Not estimated	Not estimated	Not estimated
Conclusion	Other reasons for the result	0.27 (0.02, 3.19)	5.57 (0.26, 121.27)	10.14 (0.46, 222.07)	8.8 (0.73, 106.86)
	Extension of results	1.82 (0.07, 46.63)	0.33 (0.01, 8.53)	0.59 (0.02, 15.25)	1.19 (0.05, 30.96)
	Implications	1.82 (0.07, 46.63)	0.33 (0.01, 8.53)	0.59 (0.02, 15.25)	1.19 (0.05, 30.96)
	Funding	0.57 (0.03, 9.64dd)	0.19 (0.01, 4.20)	0.35 (0.02, 7.57)	0.7 (0.03, 15.46)

The words in bold indicate significance at the 0.05 level

### Number of organizations

Table 4 highlights significant differences between there being one organization and more than one organization appearing in one item, that of personal contacts [OR = 0.05, 95%CI (0.01, 0.41)].At the same time, compared with the SWSRs that had been completed by more than one organization, 10 items (qualification, retrieval software, personal contacts, confounding, blinding, regression analysis, rationality of exclusion criteria, extension of results, implications and funding), were found to have a high rate of reporting by one organization.

### Number of authors

Table 4 shows significant differences between there being more than three authors and less than four authors under two items, namely research information [OR = 0.20, 95%CI (0.06, 0.64)] and retrieval software [OR = 684.00, 95%CI (40.49, 12,000)]. Compared with the SWSRs that had been completed by more than three authors,19 items (population, qualification, research information, electronic searches, inclusion and exclusion criteria, hand searches, language, content incompleteness, personal contacts, blinding, regression analysis, heterogeneity, model description, appropriate charts, table display, rationality of exclusion criteria, quality of included studies, other reasons for the results, extension of results, implications and funding), were found to have a high rate of reporting among fewer than four authors.

### Number of countries

Table 4 shows significant difference between a publication having emerged from one country and more than one country in terms of the following six items, strategy [OR = 33.53, 95%CI (1.60, 704.07)], electronic searches [OR = 0.00, 95%CI (0.00, 0.04)], inclusion and exclusion criteria [OR = 14.35, 95%CI (3.08, 66.93)], hand searches [OR = 0.06, 95%CI (0.01, 0.30)], blinding [OR = 0.04, 95%CI (0.00, 0.69)]and confounding [OR = 22.00, 95%CI (2.17, 223.23)]. Compared with the SWSRs that were completed in more than one country,10 items (qualification, research information, electronic searches, hand searches, blinding, model description, appropriate charts, chart display, potential biases, and funding) were found to have a high rate of reporting in reviews covering only one country.

## Discussion

Our study analyzed 57 SWSRs that were published in the Campbell Collaboration online library. After the first was published in 2004, the number of SWSRs increased in volatility from 2004 to 2017. These results indicate the increasing need for social welfare researchers to undertake secondary studies, which, in turn, highlights the significance of evidence quality. This study used the MOOSE checklist to evaluate the quality of the SWSRs published by the Campbell Library, finding some items to have been incompletely reported and that the quality of reporting needs to be improved. The majority of SWSRs were found to have major flaws in the reporting characteristics of their search strategy and methods. Only 29.8% of the included SWSRs were completely reported, according to the searcher’s information. Retrieval lies at the core of SWSRs. An experienced and qualified researcher is key to the search, with incompleteness of the searcher’s information report not being conducive to the literature acquirer nor to obtaining research information [16]. Research information (item 9) relates to comprehensively searching the relevant literature, including electronic databases, manual search documents, and grey documents. The comprehensiveness of the literature search guarantees the objective and true reliability of SWSRs. In this sense, inclusion and exclusion criteria (item 12), at 54.4%, was found to be incomplete.

Regarding statistical analysis, the UK, Denmark, USA and Norway were found to have published more SWSRs than others. Among the latter countries, SFI Campbell and the University of Oxford published the most SWSRs. Universities such as the University of Warwick, the Urban Institute, and Barts and the London School of Medicine and Dentistry were located in the center of the social network. These institutions have close links with other research institutions. Frequent communication between institutions is conducive to the development and dissemination of SWSRs, and can improve the quality of systematic reviews in a consistent manner. Regarding authors, while Trine Filges published 10 SWSRs, 83.7% of the authors identified had only participated in one SWSR, while 16.3% participated in more than one. This indicates the presence of fewer authors with high yields, that these authors were not at the center of the social network, and that there was less communication between high-yielding authors. In terms of the samples in the studies included, the population ranged from children to the elderly, and the research content ranged from material life to mental health. The Campbell reviews of social welfare may thus be said to be developing towards diversity. In most of the SWSRs, only the screening process was described, with no indication of the criteria applied for screening. The latter incurs a greater risk of bias [17, 18].

Most of the studies did not mention the impact of different language databases (item 14). While the incompleteness of different language databases can lead to the emergence of bias, the integrity of the database cannot be achieved due to the limitations of research conditions. 40.4% of the SWSRs only indicated the existence of such research and did not explain the solution. There was no clear display of data and results, nor a description of the confounding, this may incur a greater bias, making it impossible for other researchers to use these studies as high-quality evidence [19, 20]. At the same time, the year of publication, the number of organizations, authors and countries involved did not affect the reporting quality of the SWSRs. Compared with SWSRs published after 2013, the SWSRs tended to have been completed by more than one organization, three authors and more than one country, with some items having a low rate of reporting prior to 2014. Collectively, these findings imply that the requirements for systemic reviews are likely to be continuously strengthened, and that the completeness of reporting in these systematic reviews is also likely to be continuously improved by research institutions through to implementation agencies. Authors’ cooperation and more inter-agency communication are also likely to improve the quality of SWSRs, with communication between the authors and institutions continuing to be emphasized in the future.

## Conclusions

This study focused on the reporting quality of SWSRs in the Campbell Collaboration online library, using the MOOSE checklist to assess this quality. According to the above analysis and the impressions collected during the process, three measures may be recommended to improve both the Campbell reviews and non-Campbell reviews on social welfare research. Firstly, The research field is concentrated and limited, and there are no influential groups. Researchers should thus work together to strengthen communication and cooperation between authors and institutions. Secondly, all journals and organizations should strengthen their requirements pertaining to the quality of systematic reviews and improve the completeness of their reports, which could increase the reproducibility and extensibility of the research. Thirdly, the applicability of research should be enhanced, with the aim of applying SWSRs to other aspects of the social sciences as much as possible. The core aspect of social welfare is that of human manners. There are many interdisciplinary subjects, such as education, economics, and management. Fourthly, underdeveloped countries should introduce SRs into their social welfare research as soon as possible [21, 22].

Overall, our research indicates that the reporting quality of SWSRs is generally good, according to the MOOSE checklist evaluation. However, a degree of bias can also be discerned. While a few authors were found to assess publication bias, this bias can have a potential impact on the results of the researches. There exist many methods by which to evaluate publication bias, such as the funnel plot. Many authors selected the types of publication, which may be impacted on the results of their research; It is, therefore, recommended that researchers avoid this.

Our study is limited in that only one topic from the Campbell Library was included. However, since the social welfare classification encompasses most systematic reviews in the Campbell Library, this limitation is unlikely to affect the representativeness of our results. While the quality evaluation of this study was carried out independently by two evaluators, and the evaluators were trained and themselves pre-evaluated prior to conducting the evaluation, the influence of subjective factors could not be eliminated, which may have affected the objectivity of the evaluation. Overall, while the SWSRs in this research was found to have good reporting quality, at the same time it should be acknowledged that there remains room for improvement in some items.

## Data Availability

Please contact the author for data requests.

## Abbreviation

SWSRs: Social welfare systematic reviews
